# Thermomechanical Modeling of Material Flow and Weld Quality in the Friction Stir Welding of High-Density Polyethylene

**DOI:** 10.3390/polym15153230

**Published:** 2023-07-29

**Authors:** Bilal Ahmad, Fahad Almaskari, Jamal Sheikh-Ahmad, Suleyman Deveci, Kamran Khan

**Affiliations:** 1 Duncan Rogers (Engineering) Ltd., 396 Hillington Road, Glasgow G52 4BL, UK; bilal@duncanrogers.com; 2 Department of Aerospace Engineering, Khalifa University of Science and Technology, Abu Dhabi 127788, United Arab Emirates; kamran.khan@ku.ac.ae; 3 Department of Mechanical Engineering, Western New England University, Springfield, MA 01119, USA; jamal.sheikh-ahmad@wne.edu; 4 Borouge Pte. Ltd., Abu Dhabi 127788, United Arab Emirates; suleyman.deveci@borouge.com

**Keywords:** friction stir welding, thermomechanical modeling, Coupled Eulerian–Lagrangian, high-density polyethylene, material flow, void formation

## Abstract

A thermomechanical model of the friction stir welding (FSW) of high-density polyethylene (HDPE) was developed by incorporating a Coupled Eulerian–Lagrangian (CEL) approach. A Johnson Cook (JC) material model of HDPE was developed through experimentally generated strain-rate- and temperature-dependent stress strain data. Two sets of FSW process parameters with minimum and maximum weld defects were numerically modeled. The numerically calculated temperature distribution, material flow and flash and potential defects were validated and discussed with the experimental results. Tracer particles allowed to visualize the material movement during and after the tool had traversed from the specified region of the workpiece. Both numerical models presented similar maximum temperatures on the upper surface of the workpiece, while the model with high traverse speed and slow rotational speed had narrower shoulder- and heat-affected zones than the slow traverse, high rotational speed model. This contributed to the lack of material flow, hence the development of voids and worm holes in the high traverse speed model. Flash and weld defects were observed in models for both sets of process parameters. However, slow traverse, high rotational speeds exhibited smaller and lesser weld defects than high traverse, slow rotational speeds. The numerical results based on the CEL approach and JC material model were found to be in good agreement with the experimental results.

## 1. Introduction

Although friction stir welding (FSW) has been extensively researched for joining steel alloys [1,2,3] and aluminum alloys [4,5,6,7], the industrial application of polymer FSW is still under consideration due to several limitations [8,9]. These mainly include the weld strength, potential defects and optimal process parameters to obtain a good-quality weld [10,11]. Several researchers [12,13,14,15] have performed experiments on the FSW of polymers to evaluate the diverse process parameters. Sheikh-Ahmad et al. [12,13] evaluated the influence of process temperatures on the material flow and weld quality of the FSW of high-density polyethylene (HDPE). The variation in the process parameters including rotational speed, welding speed and preheating temperatures were analyzed [12]. They [12] observed that the material flow was in a downward spiral movement with the counterclockwise direction of the tool. In another publication, Sheikh-Ahmad et al. [13] investigated the thermal aspects of HDPE FSW. It was concluded that large voids primarily resulted from the flow of the molten material to the advancing side [13]. Furthermore, the traverse speed was found to be a major factor in producing weld defects [13]. 

Less literature has been found that includes the numerical modeling of the whole FSW process for polymers. The available literature includes two types of models: thermal models [12,13,16,17] considering only the heat source but no observed deformation in the workpiece and computational fluid dynamics (CFD) models [14,15,18,19,20] in which the workpiece is treated as a highly viscous non-Newtonian fluid. Sheikh-Ahmad et al. [13] developed a finite element analysis model that incorporated the required heat during the FSW of HDPE. It was concluded that the maximum temperature values were found below the shoulder and at the trailing side of the tool pin [13]. Since there was no actual deformation involved, only the numerical thermal results were discussed for their numerical model [13]. Derazkola et al. [15,19] performed a CFD analysis to examine the effects of a changing tool pin geometry on the weld quality of polymer FSW. The workpiece was modeled as a non-Newtonian fluid with viscoplastic behavior [19]. Insufficient material movement during the FSW process resulted in the formation of voids and delaminated layers in the workpiece [19]. Since FSW is a classic two-way fluid structure interaction problem, a thermomechanical model incorporated with an accurate material constitutive model is crucial for the true depiction of the process.

Various material models of HDPE have been studied in the literature [21,22,23,24,25]. The published material models either discuss a strain-rate- or temperature-dependent material model. This is mainly due to the lack of sufficient experimentally generated thermomechanical material properties of the polymer. Several researchers [26,27,28,29,30] have presented the experimentally generated stress flows for various variants of HDPE at various strain rates and temperatures. Since there is no extensive strain-rate- and temperature-dependent material data for HDPE available in the literature, the need for an analytical model is critical to simulate the behavior of the material during the FSW process.

In the present work, a thermomechanical model has been developed to evaluate the behavior of the FSW process of HDPE in terms of material flow, void formation and process temperatures. A Johnson Cook (JC) material model for HDPE was developed by using experimentally generated temperature- and strain-rate-dependent stress strain data. A Coupled Eulerian–Lagrangian (CEL) approach was utilized to precisely represent the temperature distribution, material flow and flash and defect generation during the process. The numerical results were finally compared with the experimental results.

## 2. Materials and Methods

### 2.1. Experimental Work

**Figure 1:**
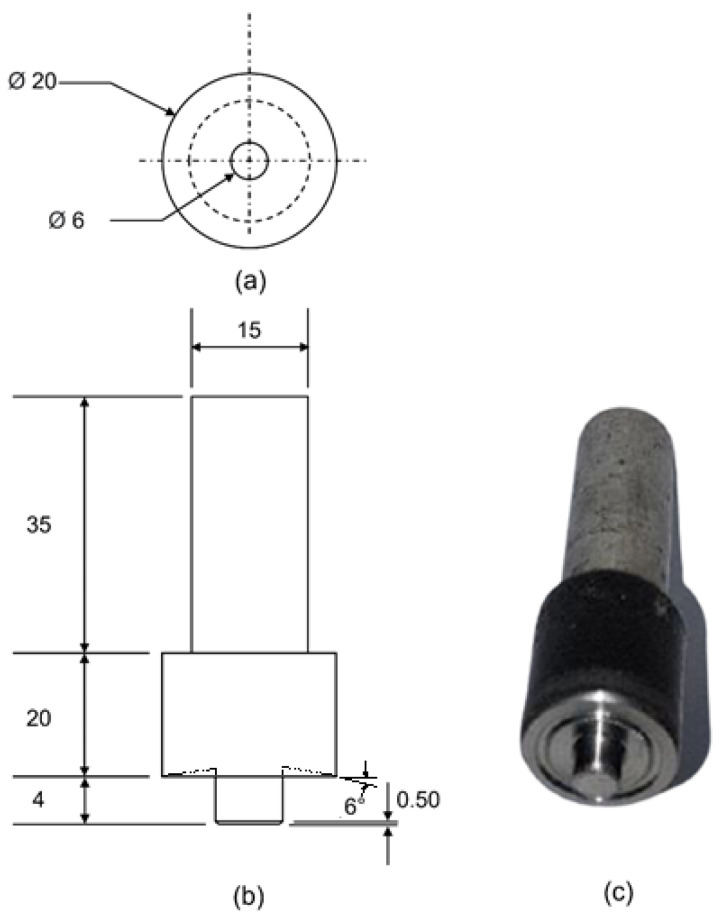
Experimental tool from the (**a**) top view, (**b**) side view, and (**c**) isometric view (all dimensions in mm).

The FSW of HDPE plates was conducted on a vertical milling machine in the butt welding configuration, as explained in a previous publication [13]. The HDPE plates of the dimensions of 160 mm × 150 mm × 5 mm were prepared by compression molding as per ISO 293:2023 [31]. Figure 1 displays the specifications of the carbon steel FSW tool. The tool shoulder is 20 mm in diameter and has a 6 mm diameter pin. A 1 mm wide and 0.5 mm deep round groove was formed with a 6° inclination on the inner surface of the tool shoulder for a smoother material flow.

**Figure 2:**
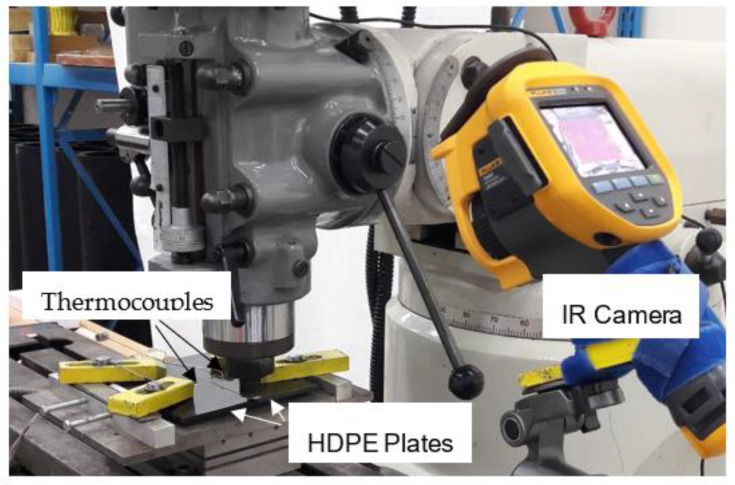
Experimental setup showing the temperature measurement using an IR camera and thermocouples.

**Figure 3:**
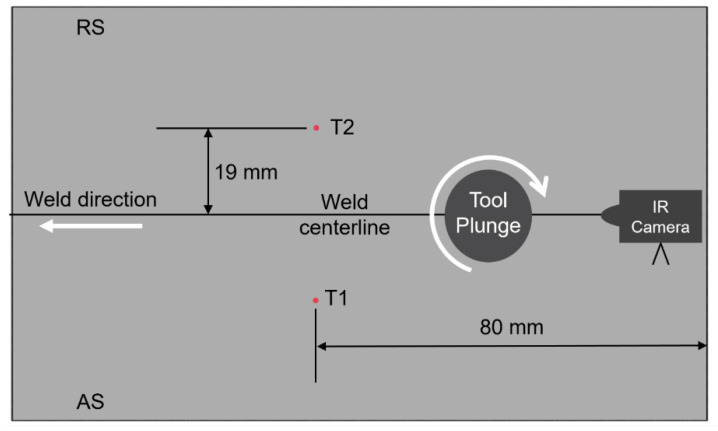
Schematic of the thermocouples’ location on the workpiece.

A diverse range of process parameters was taken into consideration to develop the design of experiments, as discussed in a previous publication [13]. The parameters being varied were the tool plunge and the traverse and rotational speeds. Three levels were varied for each of the rotational speeds and the feed, while two levels were varied for the penetration. This resulted in eighteen different combinations of factors. The welding temperatures were measured in two ways: by thermocouples and with an infrared (IR) camera placed above the top surface, as displayed in Figure 2. As the tool proceeded along the weld line, it raised the temperature of the material adjacent to it. Thus, the temperature changes were recorded by the thermocouples as well as the IR images over a period. Two blind holes were drilled 19 mm away on both sides of the weld line on the top of the workpiece to allow for placing the thermocouples. K-type thermocouples, indicated as T1 and T2 in Figure 3, were placed at specified holes around the weld line at the top surface of the workpiece. The IR camera was placed near the plunging location to track the maximum surface temperature of the HDPE behind the traversing tool [13].

**Figure 4:**
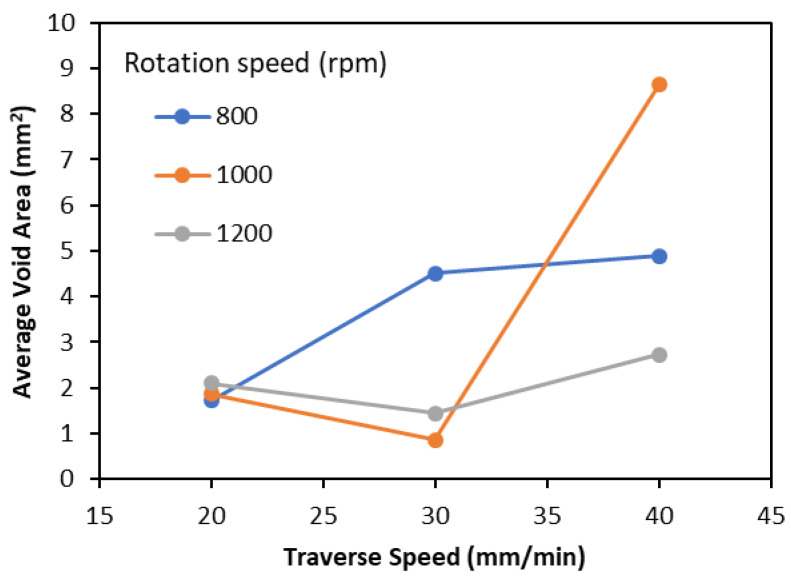
Influence of the rotational and traverse speeds on the average defect area.

The weld quality was evaluated by measuring the void content across the weld line. The void content was evaluated by measuring the void area in the cross section of the weld nugget at four different locations along the weld line [13], and an average void area was calculated. It was observed that the traverse speed had a statistically significant relationship with the average area of defects in the weld, where an increase in the traverse speed generally resulted in an increase in the average void area, as shown in Figure 4. However, the rotational speed did not exhibit a statistically significant influence on the average void area as compared to the traverse speed [13]. Therefore, two sets with extreme parameters, named Model 1 and Model 2, have been numerically modeled and discussed in the present work. Table 1 lists the process parameters that have been used for both models.

### 2.2. Numerical Modeling

#### 2.2.1. Model Description

A thermomechanical model of FSW has been developed in Abaqus/Explicit. Various models established on the CEL approach of Noh et al. [32] have reported an accurate comparison for the FSW of steel [33,34] and aluminum [35,36]. The CEL approach allows to visualize the material flow of the individual particles along with the thermal and structural variations during the FSW process. Therefore, the CEL approach has been applied in the present work. The forces in the model were calculated by solving the equation of motion (Equation (1)) in the explicit dynamics system:

(1) $$P=M\ddot{u}+C\dot{u}+Ku$$

where P is the total force acting in the system. The other three terms of Equation (1) relate to the inertial factor for any movement, the sum of all energies being dumped and the total stiffness of the body, respectively.

**Figure 5:**
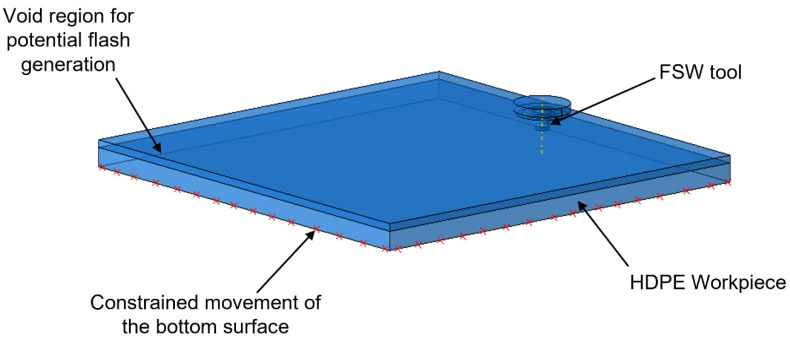
Material assignment in the workpiece based on the CEL approach.

Figure 5 illustrates the application of the CEL approach for the material assignment in the workpiece. The whole workpiece was modeled as a Eulerian body with 168,000 8-node thermally coupled EC3D8RT elements. The upper region of the workpiece was modeled with no initial material inside it. This assisted in the generation and visualization of the flash on the workpiece during and after the FSW process. Furthermore, any potential defects in the workpiece could also be observed. The FSW tool was modeled as a Lagrangian body with 21,318 8-node thermally coupled C3DRT elements. 

#### 2.2.2. Model Geometry

**Figure 6:**
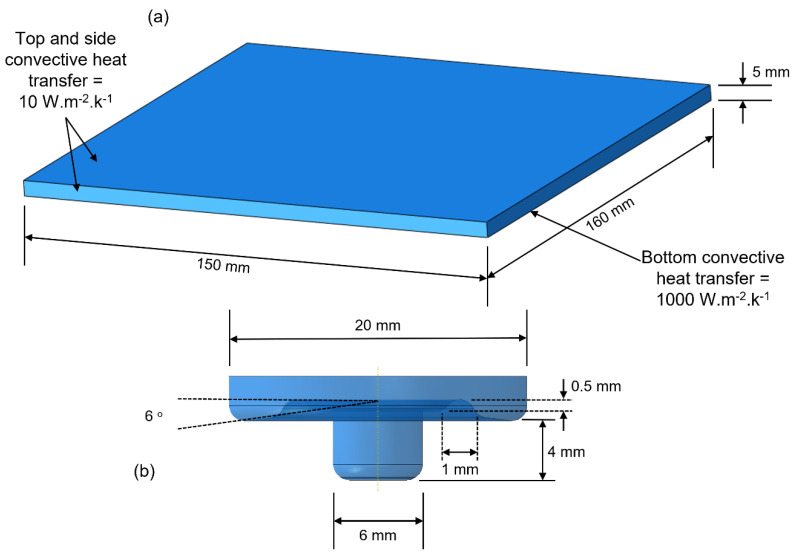
Simplified dimensions for the (**a**) workpiece and (**b**) tool.

The specifications of the HDPE workpiece and steel tool in the numerical model have been demonstrated in Figure 6. Similar to the experimental setup, the numerical tool was modeled as steel with shoulder and pin diameters of 20 mm and 6 mm, respectively. The tool pin length was 4 mm, whereas the workpiece thickness was kept as 5 mm. 

#### 2.2.3. Material Properties

The JC constitutive model is a remarkable stress-flow model to estimate the flow stresses based on the strains, strain rates and temperature-dependent changes in the process [37]. The elastic plastic JC material model is given in Equation (2).

(2) $$\sigma_{0}=\left(A+B{\bar{\varepsilon }}_{pl}^{n}\right)\left(1+Cln\frac{{\dot{\bar{\varepsilon }}}_{pl}}{{\dot{\varepsilon }}_{0}}\right)\left(1-{\left(\frac{T-T_{ref}}{T_{melt}-T_{ref}}\right)}^{m}\right)$$

where $\sigma_{0}$ refers to the yield strength of the material, ${\bar{\varepsilon }}_{pl}$ is the plastic strain, ${\dot{\bar{\varepsilon }}}_{pl}$ is the plastic strain rate and ${\dot{\varepsilon }}_{0}$ is the normalizing strain rate that is typically set at 1.0 s^−1^ [38]. *A*, *B*, *C*, *n*, *m*, $T_{melt}$ and $T_{ref}$ are the JC material model constants that are calculated using the experimentally generated temperature- and strain-rate-dependent flow stresses. 

Based on the stress strain data at diverse temperatures of 23 °C, 60 °C and 80 °C and strain rates of 0.00333 s^−1^, 0.0333 s^−1^ and 0.333 s^−1^ [39], JC parameters were developed to reflect the material behavior of HDPE during the FSW process. Table 2 presents the JC process parameters to model the material response of HDPE. The thermal and mechanical properties of HDPE are taken from [12] and are presented in Table 3. The material properties of the steel tool that has been used in this model are widely available online and are presented in Table 4. Since the steel tool is modeled as a rigid body, the mechanical response of the tool was not taken into consideration.

#### 2.2.4. Boundary Conditions

The frictional coefficient between the tool and the workpiece is crucial for the realistic depiction of heat generation between the tool and the workpiece during the process. Various researchers [40,41,42,43] have estimated different values of the frictional coefficient between polymer and metal. Mary et al. [41] calculated the static and kinetic frictional coefficient values of 0.36 and 0.23, respectively. Therefore, an average frictional coefficient value of 0.3 provided a reasonable estimation of the real process conditions in the present model.

The heat transfer from the top and side surfaces is calculated by [33]:

(3) $$q=h\left(\theta_{A}-\theta_{B}\right)+(\varepsilon \alpha \left(\theta_{A}^{4}-\theta_{B}^{4}\right))$$

where q is the total heat flux per unit area, h is the convective coefficient, ε is the emissivity coefficient, α is the Stefan–Boltzmann constant as 5.6703 × 10^−8^ W·m^−2^·K and θ is the temperature ranging from one surface to the other. The convective heat transfer values of 10 W·m^−2^·K^−1^ and 1000 W·m^−2^·K^−1^ were taken from the work of Ahmad et al. [33] on the top, sides and bottom of the workpiece, respectively. Similarly, the total energy generated during the FSW process was converted into heat, and 90% of the converted heat was assumed to be transferred into the workpiece [33].

The mesh of the model plays a significant role in determining the computational time of the whole simulation. However, a less fine mesh often results in irregular nodal contact between two separate bodies. Therefore, a high gap conduction of a 20,000 W·m^−2^·K^−1^ was introduced in the model to maintain a consistent thermal contact between the tool and the workpiece. Similarly, a general contact was implemented with an ‘all with self’ contact domain. This type of configuration ensured a reasonable contact within the workpiece itself as well [44].

Due to the high computational cost of the CEL approach, various numerical optimization techniques are assessed. Generally, there are two kind of scaling techniques: time and mass scaling [45]. The most applied mass scaling technique artificially increases the density of the material while drastically decreasing the overall simulation time [46,47]. However, Abaqus/Explicit does not allow the use of mass scaling in conjunction with the CEL approach [45], whereas the overall simulation time in the time scaling technique is reduced by increasing the relative process parameters, hence keeping the overall ratio fixed. Therefore, a time scaling of 100× was applied in the present numerical work. 

## 3. Results and Discussion

Several numerical results including temperature distribution, material flow and flash and defect generation have been calculated and compared with the experimental results. To maintain the consistency among the numerical and experimental results, the tool and workpiece geometries along with the process parameters have been kept identical.

### 3.1. Temperature Distribution

**Figure 7:**
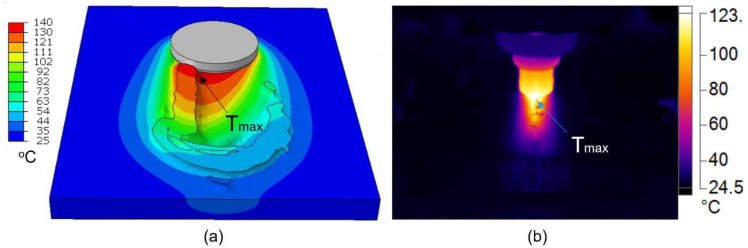
IR image of the temperature distribution behind the welding tool for welding conditions in Model 1 showing the location of the maximum surface temperature in the (**a**) numerical model and (**b**) experimental setup.

Figure 7 displays the surface temperature of the workpiece during the welding process in Model 1. It can be visualized that the weld profile is asymmetrical due to the advancing and retreating sides, also reported by various researchers [33,48]. A higher value of temperatures is found on the advancing side behind the tool. This demonstrates the significance of the thermomechanical model as compared to the commonly used thermal models. 

**Figure 8:**
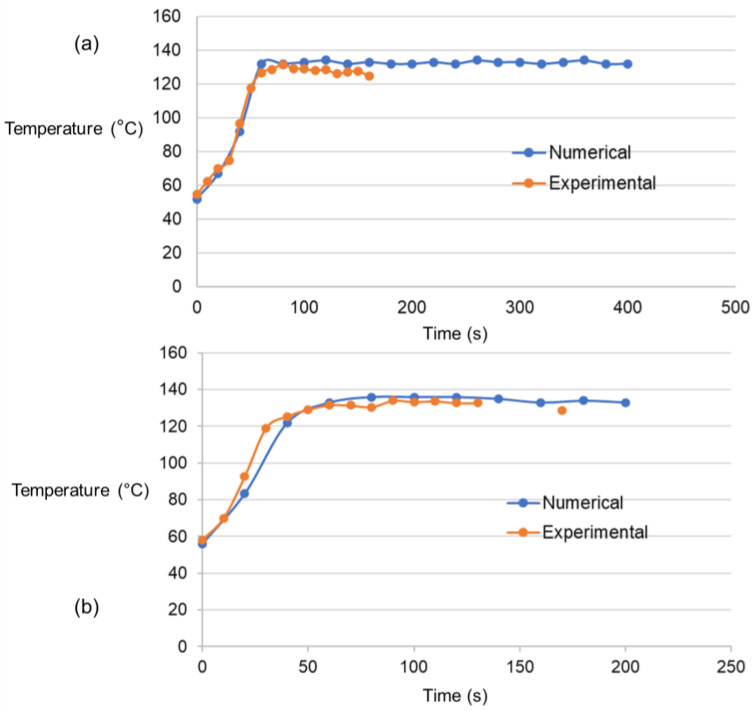
Maximum temperatures in °C behind the traversing tool in (**a**) Model 1 and (**b**) Model 2.

Figure 8 presents the maximum temperatures immediately behind the traversing tool measured by the IR camera for both models. The experimental temperatures for both models rise with time and fluctuate above and below the melting temperature, whereas the numerical-based temperatures are maintained above the melting temperature of HDPE once the process is in a steady state. It can be observed that the surge in the temperature in the beginning of the traverse stage is accurately depicted for Model 1. However, the numerical results for Model 2 show a gradual increase in the temperature for Model 2. The maximum temperatures in both models become stabilized once the process is in a steady state. A slightly higher temperature value is recorded in the numerical results as compared to the experimental results for both models. This difference can be attributed to the inconsistent locations of the flash on the workpiece in the numerical and experimental results. However, the pattern of the maximum temperatures in the numerical and experimental results is identical in both models. This demonstrates that the CEL-based numerical model with the JC material properties of HDPE could accurately depict the FSW process.

**Figure 9:**
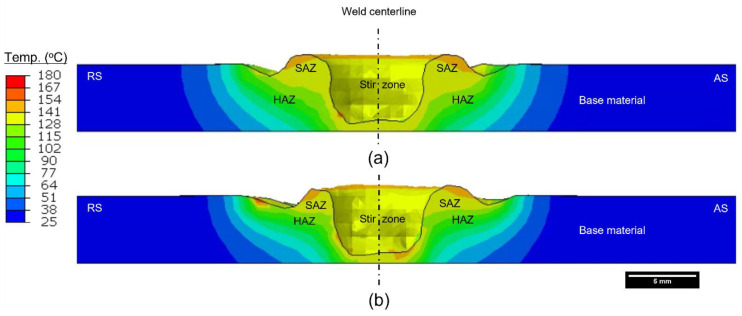
Appearance of the welded joint showing different zones: (**a**) Model 1, (**b**) Model 2 (temperature in °C).

A cross-sectional view of the welded cut is displayed in Figure 9. Based on the temperature profile and values, the shoulder-affected zone (SAZ), heat-affected zone (HAZ) and stir zone (SZ) can be easily distinguished. A higher temperature value is present on the advancing side, whereas more heat appears to be dissipated toward the sides in Model 1 than in Model 2. This demonstrates the significance of the thermomechanical model as compared to the commonly used thermal models. This also indicates that the influence of the traverse speed is crucial for an efficient joint, also reported in previous research [20,49]. The weld profiles in both models are asymmetrical due to the advancing and retreating sides, also reported by various researchers [33,48]. A relatively wider SAZ can be visualized in Model 1, whereas a narrower SAZ and HAZ are found in Model 2 due to the high traverse speed, also reported previously [33]. 

**Figure 10:**
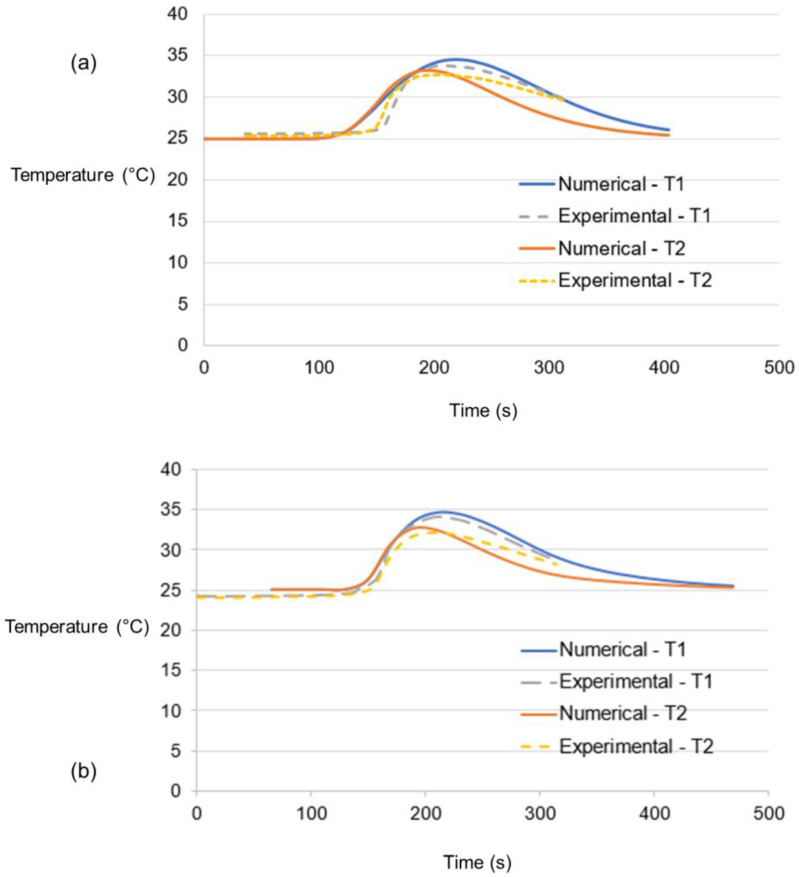
Nodal temperatures in °C on the upper surface of the workpiece in (**a**) Model 1 and (**b**) Model 2.

Figure 10 shows the temperatures recorded by both thermocouples (T1 and T2) mounted on the top surfaces of the HDPE sheets. The thermocouples’ temperatures rise as the tool approaches them and decline due to cooling as the tool moves away. This is mainly due to the heat dissipating from the top surface. In both models, it is shown that T1 is located on the advancing side of the workpiece. This explains why temperatures of T1 have higher peaks compared to T2 in both models. Furthermore, the lag in the peaks of both thermocouples’ temperatures is explained by the time that the tool comes by them, which is dependent upon their locations. A consistently identical surge is observed in the numerical- and experimental-based temperatures of both models. Similarly, the cooling-off time is also accurately depicted for T1 in both models. However, the experimental T2 of both models appears to have a slow cooling-off period compared to the numerical results. The relatively lower slope in experiments can be linked to the presence of any flash above the surface on the retreating side that allowed more heat in the vicinity of the thermocouples on the retreating side. 

### 3.2. Material Flow

**Figure 11:**
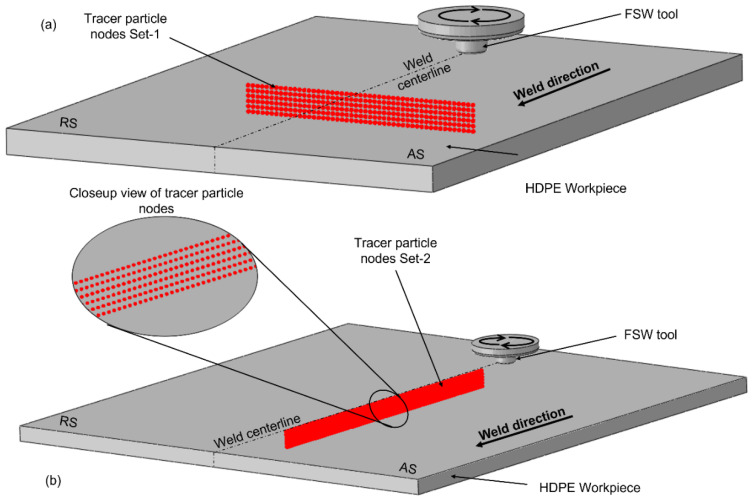
Initial location of the tracer particle nodes (**a**) Set-1 and (**b**) Set-2 in the workpiece.

**Figure 12:**
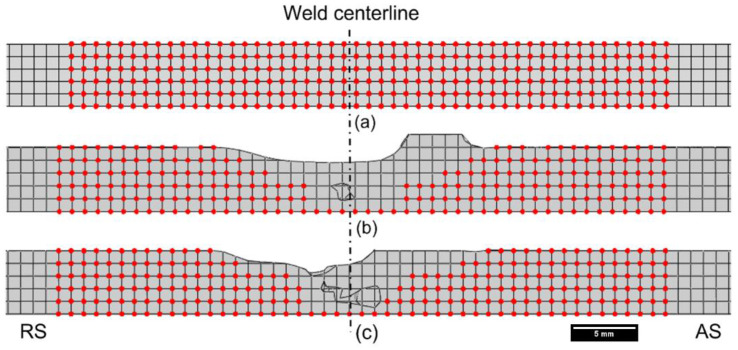
Tracer particle location (**a**) before the tool traverse, (**b**) after the tool traverse in Model 1, and (**c**) after the tool traverse in Model 2.

One of the advantages of the CEL approach is to visualize the material movement in the workpiece during the FSW process. Two tracer particle sets, named Set-1 and Set-2, were introduced in the model to track the displacement of each tracer particle during the welding, as shown in Figure 11. Figure 12a represents the cross-sectional view of the workpiece when the rotating tool has not traversed through tracer particle Set-1, whereas Figure 12b,c demonstrates the location of only those tracer particles that were present in the SZ after the tool has traversed in Model 1 and 2, respectively. A larger number of nodes were displaced in the bottom section of the stir zone in Model 1 than in Model 2 as the tool dragged the particles in the rotational direction behind it. Furthermore, both the retreating and advancing sides of Model 1 have a wider thermomechanical-affected zone as compared to Model 2. This signifies the importance of keeping high rotational and low traverse speeds for adequate material flow, hence minimizing weld defects in the workpiece. The influence of the traverse and rotational speeds on the material movement has also been highlighted by several researchers [13,50,51,52]. 

**Figure 13:**
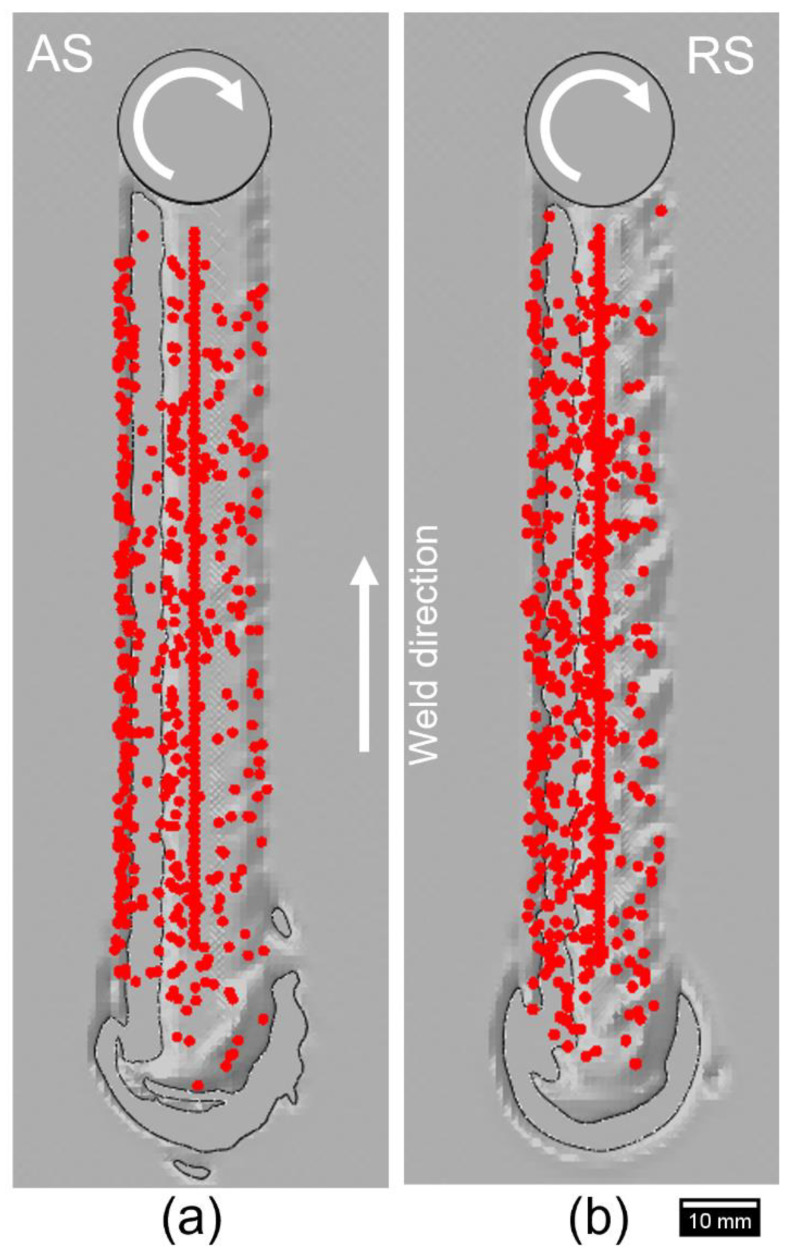
Material flow visualization with tracer particle Set-2 from the top view in (**a**) Model 1 and (**b**) Model 2.

The movement of tracer particle Set-2 from the top of the workpiece was tracked during the traverse stage and is shown for both models in Figure 13. Model 1 depicts a widely dispersed flow throughout both sides of the weld, whereas the tracer particles in Model 2 are densely located on the advancing side, only with less particles on the retreating side. This also correlates with Figure 12 as the material flow is relatively higher on the advancing side as compared to the retreating side in a specific frame. Since the temperature range is also on the higher end at the advancing side (Figure 9), the material flow is more vigorous in the specific regions as well. This establishes the significance of sufficient material flow in the SZ to achieve a good-quality weld. 

**Figure 14:**
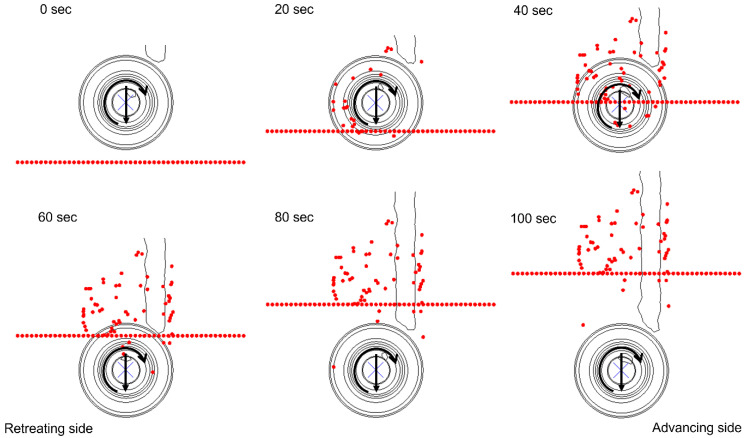
Material flow visualization in Model 1 with tracer particle Set-2 from the top view.

**Figure 15:**
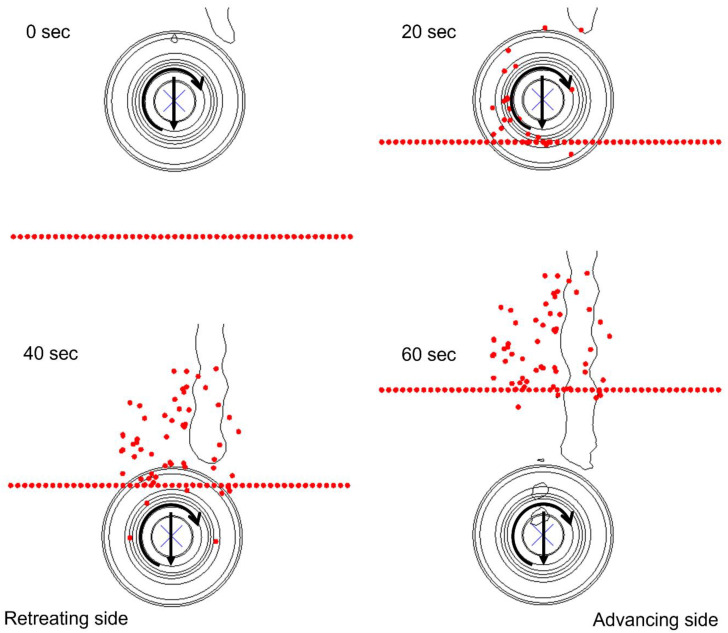
Material flow visualization in Model 2 with tracer particle Set-2 from the top view.

The material flow can be further visualized in Figure 14 and Figure 15 for Model 1 and 2, respectively, where tracer particle Set-1 is shown from the top view. The tool traverses in the weld direction with an increment of 20 s between each frame. The particles start rotating alongside the weld once they come in contact with the rotating tool. As the tool traverses, the particles also follow the weld path while maintaining the swirling pattern. The direct contact with the tool shoulder then instantly displaces the tracer particles in a rotational pattern, resulting in them moving behind the tool on the retreating side. The displacement of the particles is stopped once the material flow in that specific region of the workpiece is no longer affected by the traversing tool. It is worth noting that few tracer particles in Model 1 (Figure 14) keep on traversing with the rotating tool for an extended duration of the welding process, whereas the low rotational and high traverse speeds of the tool in Model 2 block the movement of all tracer particles once the tool traverses away from the location of Set-2, as displayed in Figure 15.

### 3.3. Flash and Potential Defect Generation

**Figure 16:**
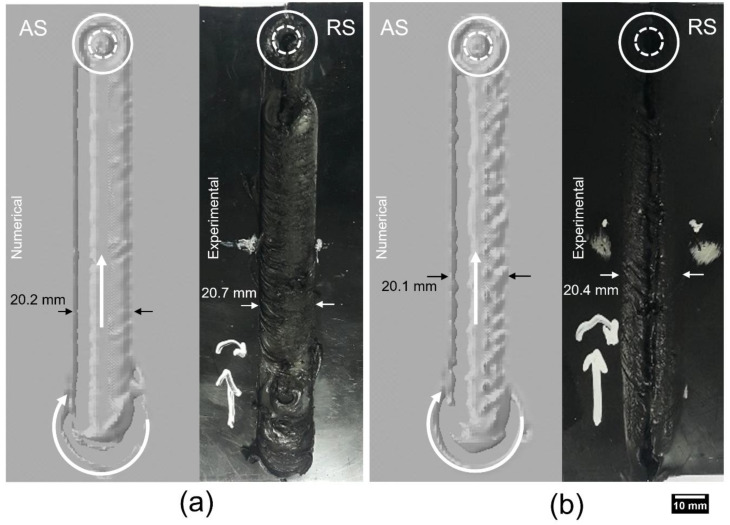
Numerical and experimental appearance of the weld bead from the top view: (**a**) Model 1, (**b**) Model 2.

The experimental and numerical results were compared for the flash and potential defect generation during the FSW process. Both numerical models exhibited a similar surface appearance with their respective experimental results, as shown in Figure 16. Major surface irregularities were observed in the unsteady region of the FSW process. Furthermore, the flash that was generated on the location of the plunge for both models (as shown in Figure 16) was later reduced once the weld had reached a steady state. Both numerical models exhibited a homogenized pattern for the flash as compared to the experimental results. However, the width of the welds in the numerical models is slightly less than the experimental results. This is because the numerical model was developed with the exact dimensions with no surface irregularities, whereas the surface irregularities along with the nonuniform thickness of the workpiece are typical in the experimental process. Therefore, the nonuniform flash generation in the experimental results can be linked to the variation in the surface quality and thickness of the workpiece.

**Figure 17:**
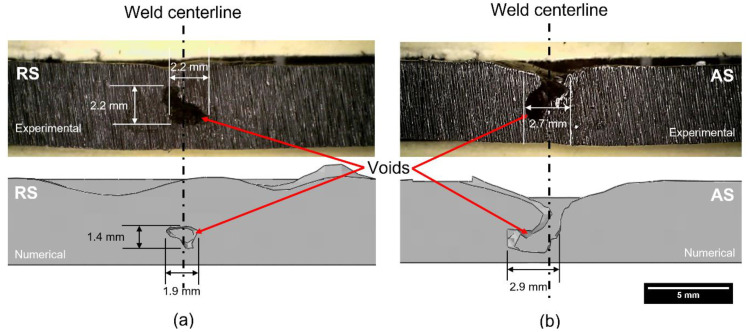
Experimental and numerical cross-sectional view of the results displaying defects in the weld: (**a**) Model 1 and (**b**) Model 2.

Voids were present in the welded region of both models, as shown in Figure 17. The location and size of the void in the numerical results can be different from the experimental results as previously explained. Sheikh-Ahmad et al. [12] mentioned that the location of the voids is independent of the advancing or retreating side as it is directly affected by the lack of sufficient temperature during the welding. It is worth noting that the defect size in numerical Model 1 is smaller than the experimental result, whereas the defect size in numerical Model 2 appears to be larger than the respective experimental result. This is because the exact location of the experimental cross-sectional cut was unknown. Since the voids are three dimensional with variable size and shape, similar weld defects from the respective models have been presented based on their shapes and sizes.

**Figure 18:**
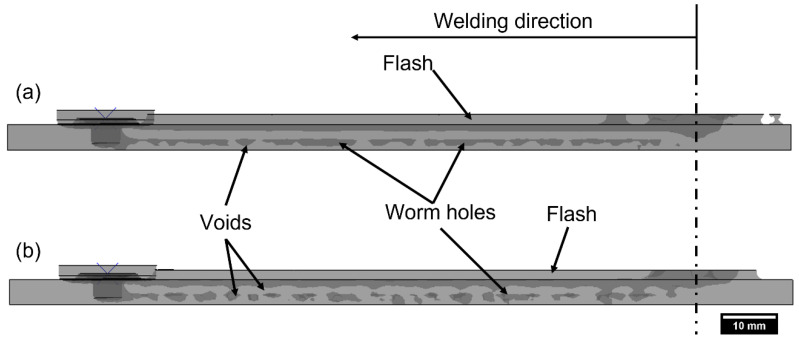
Visualization of weld defects and flash from the side view of numerical (**a**) Model 1 and (**b**) Model 2.

The internal weld defects, such as worm holes and voids, can be further visualized from the side view of the welded workpiece in Figure 18. Model 1 exhibited smaller voids and slim wormholes, whereas Model 2 had bigger voids with protruding wormholes, also reported by Sheikh-Ahmad et al. [13]. This suggests that a vigorous material flow in the FSW is accountable for the defect-free weld. In Model 2, a void was generated instantly after the tool started traversing due to insufficient temperature during the plunge stage. This signifies the dwelling stage to form a sufficient material flow for the traverse stage. Once the FSW process was in a steady state, defects started to develop throughout the traverse in both models due to the low process temperature near the tool pin tip. Therefore, optimized process parameters are required for the FSW of HDPE to obtain defect-free welds by maintaining process temperatures in the acceptable range. 

## 4. Conclusions

A thermomechanical model of the friction stir welding (FSW) of high-density polyethylene (HDPE) was developed in Abaqus/Explicit with a butt welding configuration. Two extreme sets of process parameters, 20 mm/min—1200 rpm and 40 mm/min—800 rpm welding speed and rotation speed, respectively, were experimentally and numerically generated to visualize the weld defects in the workpiece. A Couped Eulerian–Lagrangian approach was applied to calculate large deformations in the workpiece during and after the FSW process in numerical modeling. A Johnson Cook (JC) material model assisted in estimating the behavior of HDPE under high strain rates and temperatures. The numerical results, such as the temperature distribution, material flow and flash and defect generation, were verified by the experimental results. Both models illustrated similar thermal patterns on the upper surface of the workpiece. However, wider shoulder- and heat-affected zones were present in the workpiece of the 20 mm/min—1200 rpm model than the 40 mm/min—800 rpm model. This led to a more vigorous material flow in the 20 mm/min—1200 rpm model, whereas the 40 mm/min—800 rpm model had a smaller thermomechanical-affected zone. Consequently, the 20 mm/min—1200 rpm model depicted low weld defects as compared to the 40 mm/min—800 rpm model. The results obtained by the thermomechanical model in conjunction with the JC material model were in good agreement with the experimental results. Therefore, the discussed thermomechanical model of HDPE FSW can be used for optimizing the whole process along with the development of further numerical configurations, such as lap welding of dissimilar materials.

## Figures and Tables

**Table 1:** FSW process parameters for both models.

Process Parameter	Units	Model 1	Model 2
Rotational speed	rpm	1200	800
Traverse speed	mm/min	20	40

**Table 2:** JC yield strength material model properties for HDPE.

Parameter	Symbol	Unit	Value
Reference strength	*A*	MPa	4.74
Strain-hardening parameter	*B*	MPa	27.42
Strain hardening	*n*	-	0.32
Strain-rate coefficient	*C*	-	0.0591
Room temperature	$T_{ref}$	°C	23
Melting temperature	$T_{melt}$	°C	134
Temperature exponent	*m*	-	0.9439

**Table 3:** Thermal and mechanical properties of HDPE [12].

Thermal Conductivity	Yield Strength	Density	Specific Heat
W/m·°C	MPa	kg/m^3^	J/kg·°C
0.28	25	959	2250

**Table 4:** Material properties of the steel tool.

Thermal Conductivity	Thermal Expansion	Density	Modulus of Elasticity	Specific Heat
W/m·C	°C^−1^	kg/m^3^	Pa	J/(kg·°C)
16.3	1.59 × 10^−5^	8000	193 × 10^9^	490

## Data Availability

Not applicable.
